# Draft Genome Sequences of Three Clinical Isolates of the Pathogenic Yeast Candida glabrata

**DOI:** 10.1128/MRA.00278-19

**Published:** 2019-08-29

**Authors:** Pedro Pais, Mónica Galocha, Isabel M. Miranda, Acácio G. Rodrigues, Miguel C. Teixeira

**Affiliations:** a Department of Bioengineering, Instituto Superior Técnico, Universidade de Lisboa, Lisbon, Portugal; b Institute for Bioengineering and Biosciences (IBB), Biological Sciences Research Group, Instituto Superior Técnico, Lisbon, Portugal; c Department of Pathology, Division of Microbiology, Faculty of Medicine, University of Porto, Porto, Portugal; d CINTESIS-Center for Health Technology and Services Research, Faculty of Medicine, University of Porto, Porto, Portugal; University of Maryland School of Medicine

## Abstract

Here, we report the draft genome sequences of three Candida glabrata clinical isolates, 040, 044, and OL152. The isolates were recovered from patients admitted to Centro Hospitalar de S. João (CHSJ) in Porto, Portugal. Isolates 040 and 044 were taken from blood samples, while isolate OL152 was collected from urine.

## ANNOUNCEMENT

Candida glabrata, a yeast belonging to the Nakaseomyces genus from the Saccharomycetes class and Ascomycota phylum, is a major cause of candidemia worldwide (1). In this announcement, we present the draft genome sequences of three clinical isolates of C. glabrata. These draft genome sequences will contribute to our understanding of the genetic variability among C. glabrata isolates and provide clues on genomic evolution in this species. These isolates are part of a collection previously screened for azole resistance (2, 3). Isolate 044 was previously used to study azole resistance acquisition *in vitro* (4).

Isolates were grown overnight in liquid yeast extract-peptone-dextrose (YPD) medium at 30°C with orbital shaking (250 rpm) and reinoculated in fresh YPD medium until early log phase. Genomic DNA (gDNA) was extracted using the NZY microbial gDNA isolation kit (NZYTech), following the manufacturer’s instructions. Genomic DNA was sequenced on an Illumina HiSeq X platform, producing 2 × 150-bp paired-end reads. Library preparation (Nextera XT) and sequencing were carried out by Admera Health, LLC.

Illumina sequencing produced 79,811,816, 93,096,166, and 81,058,166 raw paired-end reads for isolates 040, 044, and OL152, respectively. Low-quality bases and adapters were removed using Trimmomatic (v0.38) (5). Read duplicates were removed using PRINSEQ (v0.20.4) (6). Ultimately, 66,145,112, 76,825,456, and 67,787,160 high-quality reads were used for subsequent analysis for 040, 044, and OL152, respectively.

The draft genomes were assembled into scaffolds using SPAdes (v3.12.0) (7). Scaffolds smaller than 500 bp were filtered out, and the remaining sets of scaffolds were used as draft assemblies (BioProject no. PRJNA525402). Assembly quality was analyzed using Quality Assessment Tool for Genome Assemblies (QUAST; v4.6.3) (8). The 040 assembly consists of 38.54% GC content, 202 scaffolds, a total length of 12.24 Mb, an *N*_50_ value of 400 kb, a longest scaffold of 854 kb, and an average coverage of 584×. For 044, the assembly consists of 38.51% GC content, 158 scaffolds, a total length of 12.24 Mb, an *N*_50_ value of 404 kb, a longest scaffold of 1.3 Mb, and an average coverage of 678×. For OL152, the assembly consists of 38.52% GC content, 207 scaffolds, a total length of 12.25 Mb, an *N*_50_ value of 272 kb, a longest scaffold of 1.14 Mb, and an average coverage of 583×. The assemblies align 97.6% to 98% against the reference genome (C. glabrata CBS138) (9) obtained from the *Candida* Genome Database (http://www.candidagenome.org/).

Genome annotation was performed by submitting the assemblies to the Yeast Genome Annotation Pipeline (YGAP), based on the Yeast Gene Order Browser (v7) (10). Annotation was performed specifying a post-whole-genome duplication (post-WGD) species and predicted 5,279 genes in 040, 5,278 genes in 044, and 5,287 genes in OL152.

Single nucleotide polymorphisms (SNPs) were identified against the reference genome using the Burrows-Wheeler Aligner (BWA; v0.7.17) (11) and the Genome Analysis Toolkit HaplotypeCaller (v4.0.8.1) (12). Low-quality variants were filtered out using BCFtools (v1.9) (13). The number of variants identified were 28,270, 37,158, and 37,743 for 040, 044, and OL152, respectively.

### Data availability.

This whole-genome shotgun project has been deposited in DDBJ/ENA/GenBank under the accession no. SKBI00000000, SKBJ00000000, and SKBK00000000. The versions described in this paper are the first versions, SKBI01000000, SKBJ01000000, and SKBK01000000. Raw reads are available at SRR8953802, SRR8953803, and SRR8953804.
